# Association of Fibrinogen-Like Protein 1 with Metabolic Disorders and Pregnancy Outcomes: A Case-Control Study

**DOI:** 10.2174/0118715303416614251106110927

**Published:** 2026-01-12

**Authors:** Li Jiang, Shan Zhang, Zhibiao Jiang, Xuemei Yu, Hua Jin, Peihong Chen

**Affiliations:** 1 Anhui University of Science & Technology, Huainan, 232001, China;; 2 Department of Endocrinology and Metabolism, Diabetes Ward, Fengxian Central Hospital, Shanghai, 201499, China;; 3 Department of Thoracic Surgery, Diabetes Ward, Fengxian Central Hospital, Shanghai, 201499, China

**Keywords:** Metabolic syndrome, pregnancy, fibrinogen-like protein 1, adverse pregnancy outcome, metabolic abnormility, gestational diabetes mellitus

## Abstract

**Introduction:**

This study aimed to assess the relationship between fibrinogen-like protein 1 (FGL1) and adverse pregnancy outcomes (APOs) as well as metabolic disorders during pregnancy.

**Methods:**

The pregnant women from 24 to 28 weeks of gestation were divided into different groups according to the number of metabolic abnormalities in a 2:1:1 ratio. A total of 120 cases were in the normal metabolic group, 120 cases were in the one metabolic disorder group, and 60 cases were in the two or more metabolic disorder groups. Blood collection and other sample collections were performed at 24-28 weeks of gestation.

**Results:**

Serum FGL1 levels increased gradually across the normal metabolism group, one metabolic disorder group, and the two or more metabolic disorders group (H=9.410, *P*=0.009). Serum FGL1 level was extensively higher in the gestational diabetes mellitus (GDM) group compared to the normal glucose tolerance group (*P*<0.001). Compared with 190 women without APOs, serum FGL1 was higher in 59 women who developed adverse outcomes (*P*<0.05). Receiver operating characteristic (ROC) curve analysis showed that the combination of FGL1 with traditional indicators had higher predictive performance for APOs, with the area under the AUC of 0.718, than that of traditional indicators alone (AUC=0.700).

**Discussion:**

High serum FGL1 may be a potential biomarker to monitor the severity of metabolic abnormality to enhance the predictive capability of traditional biomarkers for APOs.

**Conclusion:**

Serum FGL1 in mid-pregnancy was closely related to metabolic disorders, GDM, and APOs.

## 1 INTRODUCTION

A metabolic syndrome during gestation involves a cluster of metabolic abnormalities during pregnancy, including overweight and obesity, insulin resistance (IR), dyslipidemia, and increased blood pressure [1]. Several risk factors associated with gestational metabolic syndrome (GMS) include the following [2-4]: Overweight or obesity increases the likelihood of GMS, particularly in the abdominal region. IR, the condition by which cells throughout the body fail to respond effectively to insulin, is a major contributor to the causation of GMS. The other symptoms commonly associated with GMS are dyslipidemia, characterized by an abnormal blood lipid level, specifically high triglycerides and low high-density lipoprotein cholesterol (HDL-C). High blood pressure during pregnancy is another major risk factor. Age could be one of the factors affecting the susceptibility of GMS. Women over 35 years of age are at a higher risk. Family medicine history regarding type 2 diabetes has increased the risk for GMS. Poor dietary habits, which include high consumption of processed foods and low consumption of fruits and vegetables, have promoted the development of GMS. It has also been specified that physical inactivity finds a place among the list of recognized risks. Cigarette smoking and the use of alcohol will further heighten the risk of metabolic syndrome during pregnancy. These risk factors must be understood to facilitate early detection and management of GMS, thereby minimizing adverse maternal and neonatal outcomes.

GMS will increase the risk of adverse maternal and neonatal outcomes and the long-term risk of cardiovascular disease. It is believed that, with the improvement of life quality and changes in dietary habits, the incidence of GMS has been on the rise and seriously threatens maternal and fetal health [5]. Much more attention has been paid by the public to metabolic diseases like gestational diabetes mellitus (GDM) or GMS. Early recognition of the risk factors for GMS, understanding its metabolic changes in pregnant women, and further investigating its association with pregnancy outcomes is highly important for reducing adverse maternal and neonatal outcomes.

The gene of fibrinogen-like protein 1(FGL1) characterizes the family of fibrinogen and has promoter activities of mitochondria [6-11]. Most of its synthesis has been localized in the parenchymal cells of the liver [12]. It is considered a product of liver cell regeneration and participates in hepatocyte mitosis and energy utilization, thereby regulating the processes of lipid metabolism and blood sugar levels [8, 10, 13-15]. FGL1 expression has also been associated with hyperlipidemia, IR, and hyperglycemia [10]. During hyperlipidemic conditions, FGL1 expression was mediated by C/EBPβ-driven transcription in primary hepatocytes treated with palmitate and mice on a high-fat diet [10]. IR leads to the upregulation of FGL1; its high levels could induce IR by activating the phosphorylated JNK pathway in C2C12 myocytes [10]. Clinical studies have also indicated that circulating serum levels of FGL1 are increased in overweight/obesity, nonalcoholic fatty liver disease (NAFLD), GDM, and type 2 diabetes mellitus (T2DM) patients [16]. Glucose tolerance, insulin tolerance, and the glucose clamp technique in these subjects have been carried out; all these tests have shown overexpression of FGL1 to impair glucose utilization and lead to IR [6].

Nevertheless, there is still limited research regarding FGL1, GDM, and pregnancy outcomes. The study was conducted to study the relationship between FGL1, metabolic abnormalities during pregnancy, and adverse pregnancy outcomes (APOs).

## 2 SUBJECTS AND METHODS

### 2.1 Ethics Approval

The Ethics Committee of Fengxian Central Hospital reviewed and approved the study, and the Helsinki Declaration has been followed (Ethics Approval No. KY201604). Before enrollment, written informed consent was obtained from each subject.

### 2.2 Study Population

This research was a case-control study, and pregnant women with gestation between 24 and 28 weeks were enrolled in the study at our hospital between August and October 2018, with complete follow-up on the pregnancy outcomes. Random sampling was employed to ensure that the selected pregnant women represented the broader population.

Inclusion criteria were as follows: (1) pregnant women aged 20 to 40 years; (2) gestational age between 24 and 28 weeks; (3) complete follow-up on pregnancy outcomes; (4) willingness to participate in the study and provision of written informed consent. Exclusion criteria included the use of medications known to influence glucose or lipid metabolism; pregestational diabetes mellitus; pregestational hypertension; hepatic or renal dysfunction; autoimmune diseases; hematological disorders; tumors; thyroid diseases; cardiovascular or cerebrovascular diseases; and other conditions known to affect glucose and lipid metabolism or serum FGL1 levels.

### 2.3 GMS Classification

Blood collection and other sample collections were performed at 24-28 weeks of gestation. Four kinds of situation listed below were considered to be of GMS: (1) Pregnancy BMI ≥25 kg/m^2^; (2) GDM diagnosis; (3) TG ≥3.23 mmol/L; (4) Hypertension: Normal blood pressure before to pregnancy, but at least two times after 20 weeks of gestation, assessed four hours apart, SBP ≥140 mmHg and/or DBP ≥90 mmHg [17, 18]. According to the different metabolic components of GMS, participants were matched by age and gestational age (weeks) (GAs) in the following ratio: 2:2:1, with 120 cases in the normal metabolic group (G1), 120 cases in the one kind of metabolic disorder group (G2), and 60 cases in the two or more kind of metabolic disorders group (G3).

### 2.4 Research Methods

#### 2.4.1 Medical History and Laboratory Examinations

Maternal age, BMI, gestational age (GA), and prenatal height and weight were all considered general data. Fasting plasma glucose (FPG), 1-hour plasma glucose (1h-PG), 2-hour plasma glucose (2h-PG), fasting plasma insulin (FINS), 1-hour postprandial insulin (1h-INS), 2-hour postprandial insulin (2h-INS), glycosylated hemoglobin (HbA1c), total cholesterol (TC), triglycerides (TG), high-density lipoprotein cholesterol (HDL-c), low-density lipoprotein cholesterol (LDL-c), aspartate aminotransferase (AST), alanine aminotransferase (ALT), uric acid (UA), and creatinine (Cr) were all measured during the oral glucose tolerance test (OGTT).

An automated biochemical analyzer (DXC 800; Beckman Coulter, Brea, CA, USA) was used to measure TC, TG, LDL-c, HDL-c, ALT, AST, FPG, 1h-PG, and 2h-PG. Glycosylated hemoglobin (HbA1c) was measured using the HLC 723 G7 analyzer (Tosoh, Tokyo, Japan). The Roche Elecsys 1010 immunoassay analyzer and electrochemiluminescence immunoassay kits (Roche Diagnostics, Germany) were used to measure FINS, 1h-INS, and 2h-INS levels.

#### 2.4.2 Calculation of Related Indicators

Body Mass Index (BMI) = Weight (kg) / Height (m)^2^. The area under the ROC curve (AUC) for glucose from the 2h-OGTT: AUC for glucose = (FPG + 1h-PG) × 1/2 + (1h-PG + 2h-PG) × 1/2. AUC for insulin from the 2h-OGTT: AUC for insulin = (FINS + 1hINS) × 1/2 + (1h-INS + 2h-INS) × 1/2. The HOMA-IR = FPG × FINS / 22.5 The HOMA-β = 20 × FINS / (FPG - 3.5).

#### 2.4.3 Measurement of Serum FGL1 Concentration

Serum FGL1 concentration was quantitatively measured using an Enzyme-linked immunosorbent assay (ELISA) kit (CSB-EL008653HU; Cusabio, Inc., Wuhan) with a dilution factor of 1:100. Intra-assay CV% was below 8%, while inter-assay CV% was below 10%. Human FGL1 could be detected within a range of 23.5 to 1500 pg/ml. The kit demonstrated a high degree of sensitivity with very good specificity, due to the absence of marked cross-reactivity and interference caused by related molecules.

The adverse pregnancy outcomes (APOs) in this cohort included preterm birth, perinatal fetal distress, preeclampsia, and macrosomia. A neonate born before 37 completed weeks of gestation was classified as a preterm birth [19]. According to international criteria, preeclampsia was diagnosed when new-onset hypertension (systolic blood pressure [SBP] ≥140 mmHg or diastolic blood pressure [DBP] ≥90 mmHg) occurred after 20 completed weeks of pregnancy, accompanied by proteinuria and/or evidence of end-organ dysfunction [20].

According to the Guidelines of the Society of Obstetricians and Gynaecologists of Canada (2007), perinatal fetal distress was defined as follows:

- **Non-stress test (NST):** Fetal bradycardia (<100 bpm) or tachycardia (>160 bpm) persisting for >30 minutes; baseline variability <5 bpm for ≥80 minutes or >25 bpm persisting for >10 minutes; presence of a sinusoidal pattern; decelerations lasting ≥60 seconds or occurring late; and, for accelerations—at ≥32 weeks of gestation, fewer than 2 accelerations of >15 bpm lasting >15 seconds within 80 minutes; at <32 weeks, fewer than 2 accelerations of >10 bpm lasting >10 seconds within 80 minutes.
- **Oxytocin challenge test (OCT):** Late decelerations occurring in at least 50% of contractions.

Macrosomia was defined as a neonate weighing more than 4000 g within 1 hour after birth [21].

**Subgroup analysis:** The OGTT criteria—FPG ≥ 5.1 mmol/L, 1h-PG ≥ 10.0 mmol/L, and 2h-PG ≥ 8.5 mmol/L—were used to diagnose GDM, and any one or more of them met or exceeded the requirements (per 2013 WHO recommendations) [22]. The research subjects were grouped according to whether they were combined with GDM. Among all participants, 133 cases were classified into the GDM group. Another 133 cases with normal glucose tolerance (NGT) were matched 1:1 based on age and gestational age and assigned to the NGT group. Data from the GDM and NGT groups were then compared.

## 3 STATISTICAL ANALYSIS

IBM SPSS Statistics, version 22.0 (Armonk, NY, USA), was used for data analysis, and GraphPad Prism, version 6.02 (La Jolla, CA, USA), was used to generate the graphs. Data distribution was tested for normality. Continuous variables with a normal distribution were expressed as mean ± standard deviation (mean ± SD), while those with a skewed distribution were expressed as median and interquartile range (IQR). Independent sample t-tests were used to compare normally distributed data between two groups. One-way ANOVA followed by post hoc analysis was applied to compare three or more groups. Non-normally distributed data were analyzed using nonparametric tests. Categorical variables were compared using the Chi-square test. The correlation between FGL1 and glycolipid metabolism parameters was assessed using Pearson correlation analysis. Logistic regression analysis was performed to further identify risk factors for GDM and their associated APOs. A two-sided *P* < 0.05 was considered statistically significant.

## 4 RESULTS

### 4.1 Glyco-lipid Metabolic Parameters and Serum FGL1 Levels in Different Metabolic Groups

According to the GMS diagnostic criteria, a total of 300 participants were enrolled and divided into three groups: normal metabolism, one metabolic disorder, and two or more metabolic disorders. Age, gestational age (GA), total cholesterol (TC), and low-density lipoprotein cholesterol (LDL-c) did not differ significantly among the three groups (*P* > 0.05). However, pre-pregnancy BMI, HbA1c, FPG, 1h-PG, 2h-PG, glucose AUC, FINS, 1h-INS, 2h-INS, insulin AUC, HOMA-IR, HOMA-β, triglycerides (TG), and HDL-c differed significantly among the three groups (*P* < 0.05) (Table **1**).

Serum FGL1 levels showed a gradual increase across the normal metabolism group [18,231.43 (9,019.53–25,565.05) pg/mL], one metabolic disorder group [19,349.87 (13,008.68–26,130.34) pg/mL], and two or more metabolic disorders group [19,919.60 (15,901.96–26,391.62) pg/mL] (*P* < 0.005) (Table **1**).

### 4.2 Correlation between Serum FGL1 and Glycolipid Metabolic Parameters

**Fig. (1):**
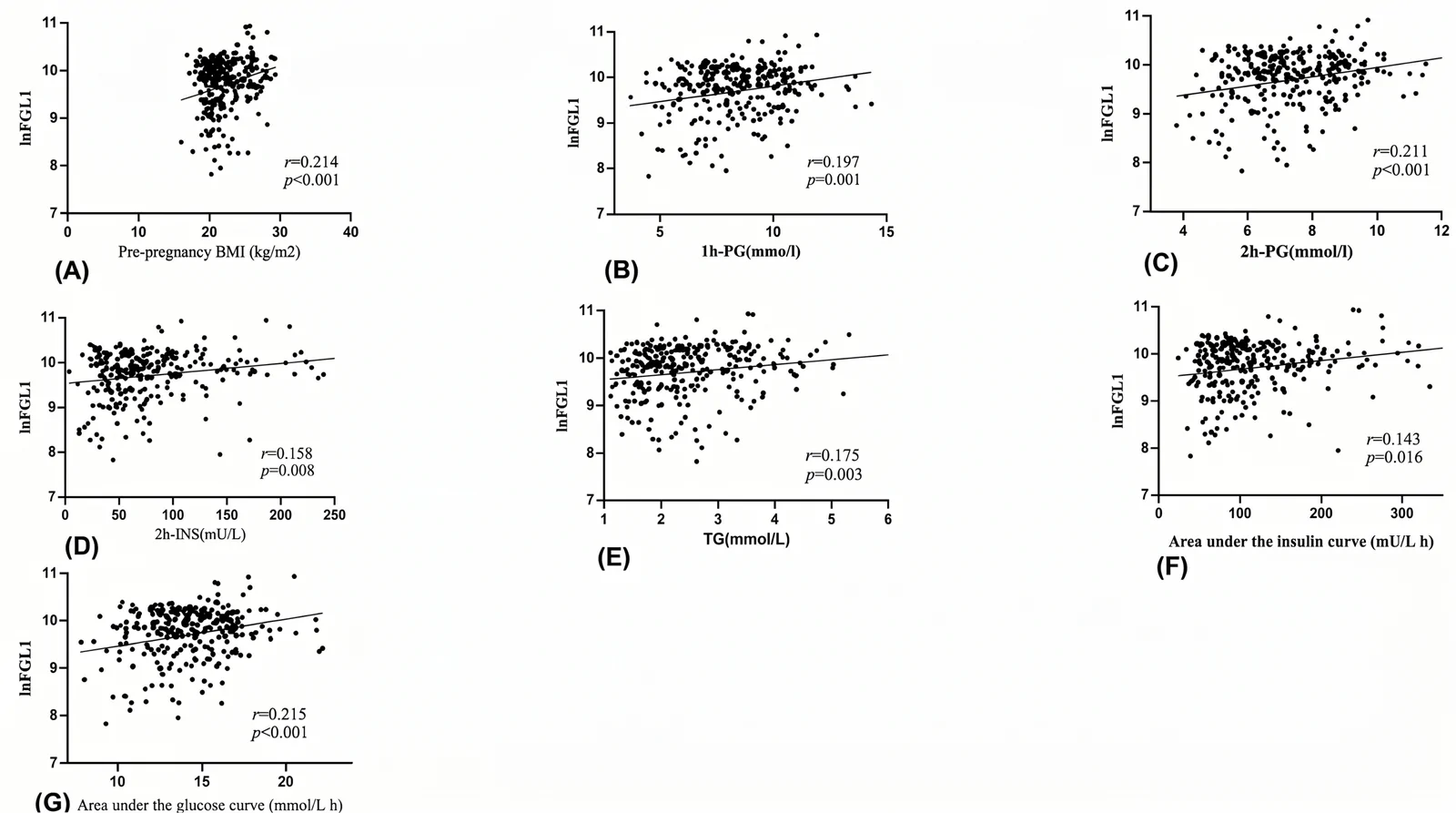
Correlation between FGL1 and different kinds of glucolipid metabolism. (**A**) FGL1 and pre-pregnancy BMI; (**B**) FGL1 and 1h-PG; (**C**) FGL1 and 2h-PG; (**D**) FGL1 and 2h-INS; (**E**) FGL1 and TG; (**F**) FGL1 and area under the insulin curve; (**G**) FGL1 and area under the glucose curve. **Abbreviations:** FGL1, fibrinogen-like protein 1; BMI, body mass index; 1h-PG, 1-hour plasma glucose; 2h-PG, 2-hour plasma glucose; 2h-INS, 2-hour plasma insulin; TG, triglycerides.

Pearson bivariate correlation analysis showed that serum FGL1 levels were positively correlated with pre-pregnancy BMI (r = 0.214, *P* < 0.001; Fig. **1A**), 1h-PG (r = 0.197, *P* = 0.001; Fig. **1B**), 2h-PG (r = 0.211, *P* < 0.001; Fig. **1C**), 2h-INS (r = 0.158, *P* = 0.008; Fig. **1D**), TG (r = 0.175, *P* = 0.003; Fig. **1E**), insulin AUC (r = 0.143, *P* = 0.016; Fig. **1F**), and glucose AUC (r = 0.215, *P* < 0.001; Fig. **1G**). There were no significant correlations between serum FGL1 levels and GA, age, FPG, HbA1c, FINS, HOMA-IR, HOMA-β, TC, HDL-c.

### 4.3 Logistic Regression Analysis of Metabolic Abnormalities

We conducted a binary logistic regression analysis with metabolic abnormalities as the dependent variable, and pre-pregnancy BMI, FPG, 1h-PG, 2h-PG, glucose AUC (AUC-G), HbA1c, FINS, 1h-INS, 2h-INS, insulin AUC (AUC-I), HOMA-IR, HOMA-β, HDL-c, TG, and FGL1 as independent variables. Among these, only pre-pregnancy BMI (OR = 1.185), FPG (OR = 4.563), TG (OR = 3.151), and FGL1 (OR = 1.791) were identified as independent risk factors for metabolic abnormalities in pregnant women (*P* < 0.05) (Table **2**).

### 4.4 Association between Serum FGL1 and Gestational Diabetes Mellitus

FPG, 1h-PG, 2h-PG, glucose AUC, HbA1c, 2h-INS, insulin AUC, and TG in the GDM group were substantially higher than those in the NGT group (*P*<0.05) (Table **3**). Moreover, serum FGL1 levels of the GDM group [19,317.03 (15,770.96, 24,832.94) pg/ml] were considerably higher than those in the NGT group [16,665.77 (9,047.53, 21,506.24) pg/ml] (*P*<0.001) (Table **3**). Binary logistic regression analysis showed that with an increase in FGL1 level, the risk of developing GDM correspondingly increased (OR = 3.275, *P* < 0.001). When GAs, age, pre-pregnancy BMI, FPG, HbA1c, insulin AUC, and TG were adjusted, FGL1 remained an independent risk factor for GDM (OR=3.080, *P*<0.001) (Table **4**).

### 4.5 Association between Serum FGL1 and APOs

Based on the presence or absence of adverse pregnancy outcomes (APOs), participants were divided into two groups: the no adverse outcomes group (n = 190) and the adverse outcomes group (n = 59). Compared with the no adverse outcomes group, the serum FGL1 level was significantly higher in the adverse outcomes group (*P* = 0.004).

Binary logistic regression analysis, adjusted for age, gestational age (GA), systolic blood pressure, pre-pregnancy BMI, FPG, HbA1c, and TG, indicated that FGL1 was an independent risk factor for adverse pregnancy outcomes (OR = 2.298, *P* = 0.043) (Table **5**).

**Fig. (2):**
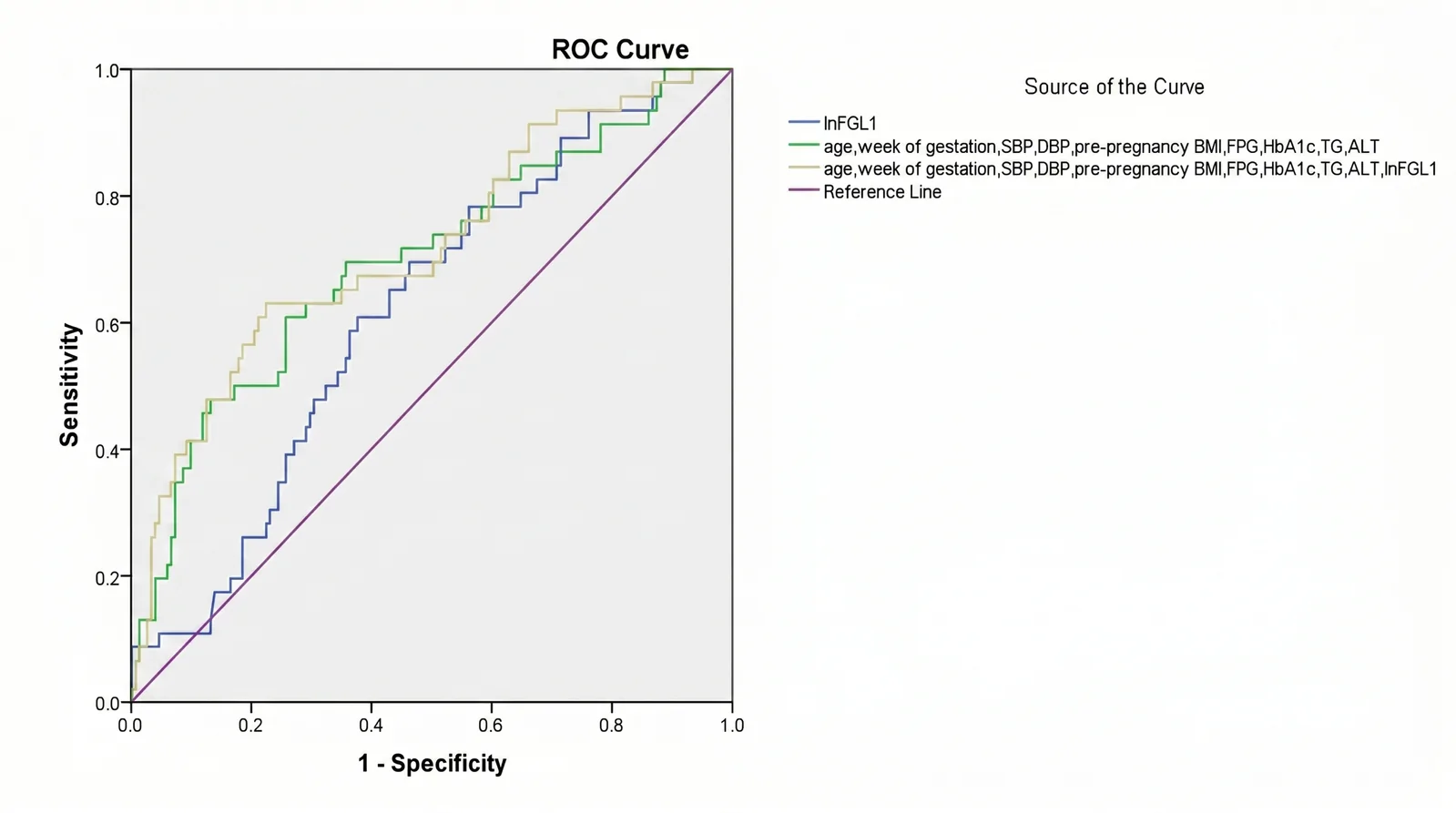
ROC curves predicting APOs in pregnant women in this study. **Note:** Model 1: FGL 1; model 2: age, week of gestation, systolic blood pressure, diastolic blood pressure, pre-pregnancy BMI, FPG, HbA1c, TG, ALT; model 3: age, week of gestation, systolic blood pressure, diastolic blood pressure, pre-pregnancy BMI, FPG, HbA1c, TG, ALT, FGL1.**Abbreviations:** BMI, body mass index; FPG, fasting glucose; HbA1c, glycosylated hemoglobin; TG, triglycerides; ALT, glutathione; FGL1, fibrinogen-like protein 1.

Receiver operating characteristic (ROC) curve analysis showed that the AUC for mid-pregnancy FGL1 was 0.618, with a sensitivity of 69.6% and a specificity of 53.6% for predicting APOs. In comparison, conventional mid-pregnancy markers, including age, GA, systolic and diastolic blood pressure, pre-pregnancy BMI, FPG, HbA1c, TG, and ALT, yielded an AUC of 0.700, with 60.9% sensitivity and 74.2% specificity. The combination of mid-pregnancy FGL1 with conventional parameters demonstrated improved predictive performance, achieving an AUC of 0.718, with a sensitivity of 63.0% and a specificity of 77.5% for predicting APOs (Fig. **2**).

## 5 DISCUSSION

This study emphasized that serum FGL1 levels in pregnant populations with metabolic abnormalities and GDM were considerably elevated. Serum FGL1 was positively correlated with pre-pregnancy BMI, 1-hour and 2-hour postprandial glucose (1h-PG and 2h-PG), the area under the glucose curve, 2h-INS, insulin area under the curve, and TG. With the increase in serum FGL1, the number and severity of metabolic abnormalities during pregnancy also increased. For every increase in the level of FGL1, the risk of adverse pregnancy outcome increased by 2.298 times. FGL1 also enhanced the predictive capability for APOs in mid-pregnancy in combination with variables including age, GAs, systolic and diastolic blood pressure, pre-pregnancy BMI, FPG, HbA1c, TG, and ALT.

Analysis of the metabolic components of GMS indicated that FGL1 levels are strongly associated with pre-pregnancy BMI, blood glucose, and blood lipids, but show no significant association with blood pressure, consistent with the findings of Wu *et al*. [16]. FGL1 levels were significantly higher in individuals with T2DM and GDM compared with controls [16]. Moreover, individuals with newly diagnosed type 2 diabetes, impaired fasting glucose, and impaired glucose tolerance exhibited elevated serum FGL1 levels [6]. These observations align with previous studies showing that serum FGL1 positively correlates with 1h-PG, 2h-PG, glucose AUC, 2h-INS, and insulin AUC, suggesting that elevated blood glucose may stimulate hepatic secretion of FGL1 [7].

In HepG2 cells, high glucose levels induced FGL1 expression *via* the signal transducer and activator of transcription 3 (STAT3) and liver cell nuclear factor pathways. Accumulation of FGL1 further promoted superoxide dismutase 1 (SOD1) expression through extracellular signaling pathways, which reduced ROS production induced by ethyl acetate and improved hepatocyte viability [7]. FGL1-deficient mice exhibited increased body weight, abnormalities in lipid metabolism, elevated fasting blood glucose, and morphological changes in white and brown adipose tissues [23, 24], suggesting that FGL1 may influence glucose and lipid metabolism *via* brown adipose tissue.

Wu *et al*. demonstrated that FGL1 enhanced insulin resistance (IR) in HepG2 cells through a hepatocyte-dependent extracellular signal-regulated kinase (ERK)1/2 pathway [4, 10]. IR was also addressed in ob/ob mice and high-fat diet mice by modest overexpression of FGL1 [6]. In pregnant women, insulin sensitivity reportedly declines by 50% to 60% with advancing gestational age [25, 26]. The positive correlations observed in our study between mid-pregnancy serum FGL1 levels and 2h-INS and insulin area under the curve, but not with HOMA-IR or HOMA-β, may reflect differences in the metabolic status of the study population.

FGL1 has been positively associated with BMI in nonpregnant populations, but no significant relationship has been observed with TG, TC, HDL-c, or LDL-c levels [27]. Pre-pregnancy BMI is strongly linked not only to pregnancy complications and offspring obesity but also to gestational weight gain. Excessive FGL1 promotes the expression of genes involved in lipid biosynthesis *via* ERK1/2–c/EBPβ–dependent signaling in both liver and adipose tissue [11, 27, 28]. In GDM patients, inflammatory factors inhibit the estrogen-mediated upregulation of ATGL through the ERα receptor, reducing triglyceride breakdown in adipocytes and further aggravating lipid metabolism disorders [29].

The present study further confirmed that, in mid-pregnancy, serum FGL1 was positively correlated with pre-pregnancy BMI and TG. Higher FGL1 levels were associated with an increased risk of adverse pregnancy outcomes (APOs). FGL1 can be integrated with age, gestational age, systolic and diastolic blood pressure, pre-pregnancy BMI, FPG, HbA1c, TG, and ALT to predict the occurrence of APOs, and its inclusion enhances the predictive power of this model, likely reflecting its role in glucose and lipid metabolism.

FGL1 also promotes trophoblast proliferation *via* the ERK1/2 signaling pathway and inhibits the regulatory effects of insulin on phosphoenolpyruvate carboxykinase expression and hepatic glucose output in HepG2 cells. Elevated placental FGL1 levels disrupt glucose utilization in the liver and muscle, thereby impairing insulin action [30, 31].

Therefore, FGL1 may be closely associated with the impairment of insulin function in the development of GDM [30]. However, its detailed mode of action needs further investigation to be ascertained. Overexpression of FGL1 robustly inhibits the pathogenesis of preeclampsia. In a study involving both mouse models and human participants, the administration of FGL1 reduced maternal hypertension and proteinuria, downregulated the levels of proinflammatory cytokines, and maintained the balance of antiangiogenic and proangiogenic substances within the placenta [32]. In addition, FGL1, which is important in hepatocyte proliferation, modification of the tumor microenvironment, and regulation of lipid metabolism, is essential for liver regeneration and in diseases such as NAFLD and liver cancer [33]. Although targeting FGL1 may be beneficial in the treatment of liver diseases and may also improve pregnancy outcomes, it is essential to understand the functions and mechanisms of FGL1 in developing effective therapeutic approaches. Much more research is needed to unravel the specific mechanisms through which FGL1 works and its full therapeutic potential.

However, this study has some limitations. First, retrospective analysis can only demonstrate an association, not a definite causal relationship, between FGL1 and GMS or APOs. Further investigation of the underlying mechanisms is needed. Second, the samples were from a single center, and the limited sample size may not accurately represent the general population. Larger sample sizes from multiple centers are needed to further confirm whether FGL1 can be used as an early predictor for GMS and APOs. Third, this study did not include sociodemographic characteristics, such as economic conditions, educational status, age, or other factors, including parity. Sociodemographic characteristics play a very important role in gestational conditions and should be analyzed in further studies. Moreover, Mendelian randomization could be performed between GMS, APOs, and FGL1 using available databases to more clearly investigate differences across population groups.

## CONCLUSION

In conclusion, mid-pregnancy serum FGL1 may be a useful biomarker reflecting the severity of glucose and lipid metabolism disorders during pregnancy and the risk of APOs. Early intervention in women with higher mid-pregnancy FGL1 levels may help reduce the incidence of APOs.

## AUTHORS’ CONTRIBUTIONS

The authors confirm their contributions to the paper as follows: writing - original draft preparation: ZJ; data collection: LJ; analysis and interpretation of results: SZ; conceptualization: XY; methodology: HJ; writing - reviewing and editing: PC. All authors reviewed the results and approved the final version of the manuscript.

## Figures and Tables

**Table 1:** Clinical indicators and baseline FGL1 characteristics by metabolic group.

**Indicators**	**Normal Metabolic Group**	**One Metabolic Disorder Group**	**Two or More Metabolic** **Disorders Group**	**Statistic**	***P* Value**
Number of cases (cases)	120	120	60	-	-
Age (years)	29.58±3.66	29.90±3.74	30.00±3.80	F*=0.348	0.706
Gestational age (weeks)	25.26±1.08	25.35±1.04	25.50±0.95	F=1.056	0.349
Pre-pregnancy BMI (kg/m^2^)	21.11±1.81	22.15±2.89	24.41±2.66	F=35.042	<0.001
FPG (mmol/L)	4.21±0.34	4.47±0.64	4.91±0.75	F=29.952	<0.001
1h-PG (mmol/L)	7.20±1.41	8.86±1.80	9.59±1.79	F=51.558	<0.001
2h-PG (mmol/L)	6.53±0.98	7.57±1.44	8.51±1.53	F=48.681	<0.001
Area under the glucose curve (mmol/Lh)	12.50±1.80	14.80±2.27	16.16±2.36	F=67.043	<0.001
HbA1c (%)	4.79±0.42	4.92±0.35	5.12±0.37	F=14.361	<0.001
FINS (mU/L)	6.37(4,70,8.97)	6.52(4.46,9.74)	11.07(8.01,15.45)	H^#^=40.285	<0.001
1h-INS (mU/L)	55.41(39.07,86.61)	66.07(43.24,88.49)	90.41(62.24,125.50)	H=17.700	<0.001
2h-INS (mU/L)	55.06(35.80,78.32)	66.96(46.58,95.15)	98.36(70.29,161.70)	H=42.428	<0.001
Area under the insulin curve (mU/L h)	88.30(68.09,129.00)	101.53(70.50,133.75)	148.52(104.51,207.77)	H=29.682	<0.001
HOMA-IR	1.21(0.84,1.77)	1.36(0.84,1.86)	2.42(1.66,3.19)	H=48.678	<0.001
HOMA-β	198.58(130.37,277.83)	164.45(97.63,237.83)	184.94(123.29,239.20)	H=8.113	0.017
TG (mmol/L)	1.94(1.61,2.32)	2.22(1.79,2.80)	3.49(3.06,4.34)	H=81.652	<0.001
TC (mmol/L)	6.30±1.07	6.11±1.06	6.06±1.02	F=1.444	0.238
LDL c (mmol/L)	3.00±0.81	2.96±0.76	2.74±0.79	F=2.317	0.100
HDL c (mmol/L)	1.76±0.27	1.70±0.30	1.58±0.28	F=8.085	<0.001
FGL1 (pg/ml)	18231.43(9019.53,25565.05)	19349.87(13008.68,26130.34)	19919.60(15901.96,26391.62)	H=9.410	0.009

**Abbreviations:** FPG, fasting glucose; 1h-PG, 1-hour plasma glucose; 2h-PG, 2-hour plasma glucose; HbA1c, glycosylated hemoglobin; FINS, fasting plasma insulin; 1h-INS, 1-hour plasma insulin; 2h-INS, 2-hour plasma insulin; HOMA-IR, Homeostasis Model Assessment of Insulin Resistance index; HOMA-β, β-cell index for homeostasis model assessment of secretion; TG, triglycerides; TC, total cholesterol; LDL-c, low-density lipoprotein; HDL-c, high-density lipoprotein; FGL1, fibrinogen-like protein 1. **Note:** *F: ANOVA; ^#^H: Chi-square test.

**Table 2:** Binary logistic regression analysis of metabolic abnormalities.

**Indicators**	**B**	**SE**	**OR(95%CI)**	***P* Value**
Age (years)	-0.038	0.040	0.963(0.889,1.042)	0.345
Gestational age (weeks)	0.090	0.153	1.095(0.811,1.478)	0.555
Pre-pregnancy BMI (kg/m^2^)	0.170	0.060	1.185(1.053,1.334)	0.005
FPG (mmol/L)	1.518	0.356	4.563(2.269,9.176)	<0.001
TG(mmol/L)	1.148	0.229	3.151(2.014,4.932)	<0.001
FGL1 (pg/ml)	0.583	0.257	1.791(1.083,2.963)	0.023

**Abbreviations:** FPG, fasting glucose; TG, triglycerides; FGL1, fibrinogen-like protein 1. B: regression coefficient; SE: standard error; OR: odds ratio; CI: confidence Interval.

**Table 3:** Baseline characteristics of NGT and GDM groups.

**Indicators**	**NGT (n=133)**	**GDM (n=133)**	**t/U**	***P* Value**
Age (years)	29.81±3.85	29.93±3.50	-0.283	0.777
Gestational age (weeks)	25.26±1.01	25.39±0.99	-1.075	0.283
Pre-pregnancy BMI (kg/m^2^)	21.95±2.53	22.32±2.79	-1.134	0.258
Systolic blood pressure (mmHg)	113.02±10.17	110.80±11.62	1.651	0.100
Diastolic blood pressure (mmHg)	71.73±7.18	70.47±8.32	1.326	0.186
FPG (mmol/L)	4.23±0.35	4.73±0.76	-6.796	<0.001
1h-PG (mmol/L))	7.27±1.43	9.60±1.70	-12.112	<0.001
2h-PG (mmol/L)	6.57±0.98	8.26±1.52	-10.596	<0.001
Area under the blood glucose curve (mmol/L h)	12.64±1.83	15.98±2.14	-13.577	<0.001
HbA1c (%)	4.87±0.34	4.99±0.37	-2.932	0.004
FINS (mU/L)	7.32(4.98,10.27)	7.27(4.61,11.64)	8363	0.723
1h-INS (mU/L)	60.00(41.53,93.48)	68.97(44.11,102.10)	7717	0.132
2h-INS (mU/L)	60.79(38.37,85.29)	72.90(50.54,115.60)	6345	<0.001
Area under the insulin curve (mU/L h)	92.24(70.27,139.13)	110.39(81.30,166.59)	7120	0.013
HOMA-IR	1.42(0.93,1.90)	1.57(0.93,2.65)	7598	0.109
HOMA-β	205.89(130.79,292.74)	137.54(96.88,211.56)	5690	<0.001
TG (mmol/L)	2.17(1.68,2.62)	2.50(1.83,3.33)	6937	0.003
TC (mmol/L)	6.27±1.09	6.15±1.04	0.948	0.344
LDL C (mmol/L)	2.74(2.42,3.46)	2.86(2.37,3.39)	8585	0.756
HDL C (mmol/L)	1.72(1.52,1.93)	1.69(1.46,1.88)	8010	0.218
FGL1 (pg/ml)	16665.77(9047.53,21506.24)	19317.03(15770.96,24832.94)	6147	<0.001

**Abbreviations:** NGT, normal glucose tolerance; GDM: gestational diabetes mellitus; FPG, fasting glucose; 1h-PG, 1-hour plasma glucose; 2h-PG, 2-hour plasma glucose; HbA1c, glycosylated hemoglobin; FINS, fasting plasma insulin; 1h-INS, 1-hour plasma insulin; 2h-INS, 2-hour plasma insulin; HOMA-IR, the Homeostasis Assessment of Insulin Resistance index; HOMA-β, Homeostasis Model Assessment of β-cell Secretion index; TG, triglycerides; TC, total cholesterol; LDL-c, low-density lipoprotein; HDL-c, high-density lipoprotein; FGL1, fibrinogen-like protein 1.

**Table 4:** Logistic regression analysis of FGL1 and GDM.

**Indicators**	**Univariate Analysis**				**Multivariate Analysis**			
	B	SE	OR (95%CI)	*P* Value	B	SE	OR (95%CI)	*P* Value
Week of pregnancy (weeks)	0.132	0.123	1.142(0.897,1.453)	0.283	0.164	0.157	1.179(0.866,1.605)	0.296
Age (years)	0.010	0.033	1.010(0.945,1.078)	0.776	-0.016	0.041	0.984(0.908,1.066)	0.688
Pre-pregnancy BMI (kg/m^2^)	0.053	0.047	1.054(0.962,1.155)	0.257	-0.124	0.063	0.884(0.781,1.000)	0.051
FPG (mmol/L)	2.027	0.341	7.588(3.889,14.803)	<0.001	2.092	0.401	8.100(3.693,17.767)	<0.001
1h-PG(mmol/L)	0.933	0.114	2.541(2.031,3.180)	<0.001	-	-	-	-
2h-PG (mmol/L)	1.024	0.133	2.783(2.143,3.614)	<0.001	-	-	-	-
Area under the glucose curve (mmol/L h)	0.936	0.114	2.549(2.039,3.187)	<0.001	-	-	-	-
HbA1c (%)	1.048	0.370	2.851(1.379,5.893)	0.005	0.132	0.505	1.141(0.425,3.068)	0.793
2h-INS (mU/L)	0.011	0.003	1.011(1.006,1.017)	<0.001	-	-	-	-
Insulin area under the curve (mU/L h)	0.006	0.002	1.006(1.002,1.010)	0.006	0.002	0.002	1.002(0.997,1.006)	0.541
HOMA-IR	0.258	0.110	1.294(1.043,1.606)	0.019	-	-	-	-
TG (mmol/L)	0.476	0.141	1.609(1.22,2.123)	0.001	0.322	0.165	1.380(0.998,1.908)	0.051
FGL1 (pg/ml)	1.186	0.265	3.275(1.948,5.506)	<0.001	1.125	0.318	3.080(1.653,5.738)	<0.001

**Abbreviations:** GDM: gestational diabetes mellitus; FPG, fasting glucose; 1h-PG, 1-hour plasma glucose; 2h-PG, 2-hour plasma glucose; HbA1c, glycosylated hemoglobin; 2h-INS, 2-hour plasma insulin; HOMA-IR, Homeostasis Assessment of Insulin Resistance index; TG, triglycerides; FGL1, fibrinogen-like protein 1. B: regression coefficient; SE: standard error; OR: odds ratio; CI: confidence interval.

**Table 5:** Binary logistic regression analysis of APOs.

**Model 1**			**Model 2**			**Model 3**		
**Indicator**	OR(95%CI)	*P* Value	Indicators	OR (95%CI)	*P* Value	Indicators	OR (95%CI)	*P* Value
**FGL1**	2.366 (1.266,4.424)	0.007	Age (years)	1.023(0.933,1.122)	0.625	Age (years)	1.013(0.922,1.114)	0.782
-	-	-	Gestational age (weeks)	0.877(0.612,1.257)	0.476	Gestational age (weeks)	0.841(0.578,1.222)	0.363
-	-	-	Systolic blood pressure (mmHg)	1.060(1.017,1.106)	0.006	Systolic blood pressure (mmHg)	1.051(1.005,1.099)	0.030
-	-	-	Diastolic blood pressure (mmHg)	1.001(0.948,1.057)	0.965	Diastolic blood pressure (mmHg)	1.005(0.948,1.064)	0.877
-	-	-	Pre-pregnancy BMI (kg/m^2^)	1.088(0.951,1.245)	0.220	Pre-pregnancy BMI (kg/m^2^)	1.067(0.929,1.226)	0.356
-	-	-	FPG (mmol/L)	1.373(0.843,2.237)	0.203	FPG (mmol/L)	1.361(0.823,2.250)	0.229
-	-	-	HbA1c(%)	2.066(0.694,6.157)	0.193	HbA1c(%)	2.063(0.681,6.253)	0.201
-	-	-	TG (mmol/L)	0.946(0.660,1.356)	0.763	TG (mmol/L)	0.918(0.630,1.338)	0.658
-	-	-	ALT (µl)	0.993(0.974,1.012)	0.459	ALT (µl)	0.992(0.974,1.012)	0.436
-	-	-	-	-	-	FGL1	2.298(1.028,5.141)	0.043

**Abbreviations:** APOs, adverse pregnancy outcomes; FPG, fasting glucose; HbA1c, glycosylated hemoglobin; TG, triglycerides; ALT, glutathione; OR: odds ratio; CI: confidence interval.

## Data Availability

All data generated or analyzed during this study are included in this published article.

## STANDARDS OF REPORTING

STROBE guidelines were followed.
